# Metachronous early gastric cancer over a period of 13 years after eradication of *Helicobacter pylori*

**DOI:** 10.1007/s12328-014-0536-9

**Published:** 2014-11-04

**Authors:** Atsushi Mitsunaga, Tomoko Tagata, Tetsuya Hamano, Honami Teramoto, Motoyasu Kan, Yutaka Mitsunaga, Maki Tobari, Izumi Shirato, Miho Shirato, Shuhei Yoshida, Masahiko Shimada, Takayoshi Nishino

**Affiliations:** 1 Department of Endoscopy, Tokyo Women’s Medical University Yachiyo Medical Center, 477-96 Owada-Shinden, Yachiyo, Chiba 276-8524 Japan; 2 Department of Gastroenterology, Tokyo Women’s Medical University Yachiyo Medical Center, 477-96 Owada-Shinden, Yachiyo, Chiba 276-8524 Japan

**Keywords:** *Helicobacter pylori*, Eradication, Early gastric cancer, Metachronous cancer, Endoscopic treatment

## Abstract

Stomach cancer can occur during chronic inflammation from *Helicobacter pylori* (*HP*) infection, and its occurrence can be suppressed by eradication of *HP*. However, the effects of suppressing stomach cancer by *HP* eradication are limited, and the cancer is known to recur even after eradication of this infection. Here, we report the case of a 56-year-old male patient with gastric cancer who, although undergoing *HP* eradication after treatment of early gastric cancer with endoscopy, experienced five metachronous cancer recurrences over a period of 13 years. Whether observation of patients who undergo eradication of *HP* due to peptic ulcers or chronic gastritis and patients who undergo eradication after endoscopic treatment for early gastric cancer should be performed at the same interval is an issue that must be addressed in the future. The appropriate observation period for each patient must be established while considering the burdens to the patient and from the medical economic perspective.

## Introduction

Eradication of *Helicobacter pylori* (*HP*) after endoscopic treatment of early gastric cancer significantly reduces the occurrence of metachronous early gastric cancer [1–3]. In 2010, eradication of *HP* after endoscopic treatment of early gastric cancer was approved under the Japanese system of health insurance based on results that the onset of metachronous early gastric cancer could be controlled by *HP* eradication [1]. However, eradication of *HP* does not completely suppress the occurrence of metachronous gastric cancer, and some cases of recurrence after eradication have been reported [4].

Here, we report the case of a patient experiencing 5 occurrences of metachronous gastric cancer during 13 years of observation after endoscopic treatment of primary early gastric cancer and eradication of *HP*.

## Case report

In 1998, a 56-year-old male was first diagnosed with early stage gastric cancer. In March 1998, his previous doctor removed a Stage IIa early gastric tumor (12 mm) from the lesser curvature of the antrum via endoscopic mucosal resection (EMR). Postoperatively, the patient tested positive for *HP* infection. Eradication was performed in October 1998 and verified to be successful by *HP* cultures and microscopy.

The patient underwent periodic check-ups, and no recurrence of gastric cancer or metachronous early gastric cancer was found. Eight years later, in October 2006, a Stage IIa + IIc early gastric tumor (15 mm) was detected at the lesser curvature of the antrum, and a Stage IIa early gastric tumor (10 mm) at the posterior wall of the antrum. They were again removed via EMR by his previous doctor, and were classified as m, tub1, HM0, VM0, ly(−), v(−) according to the histopathology findings.

**Fig. 1:**
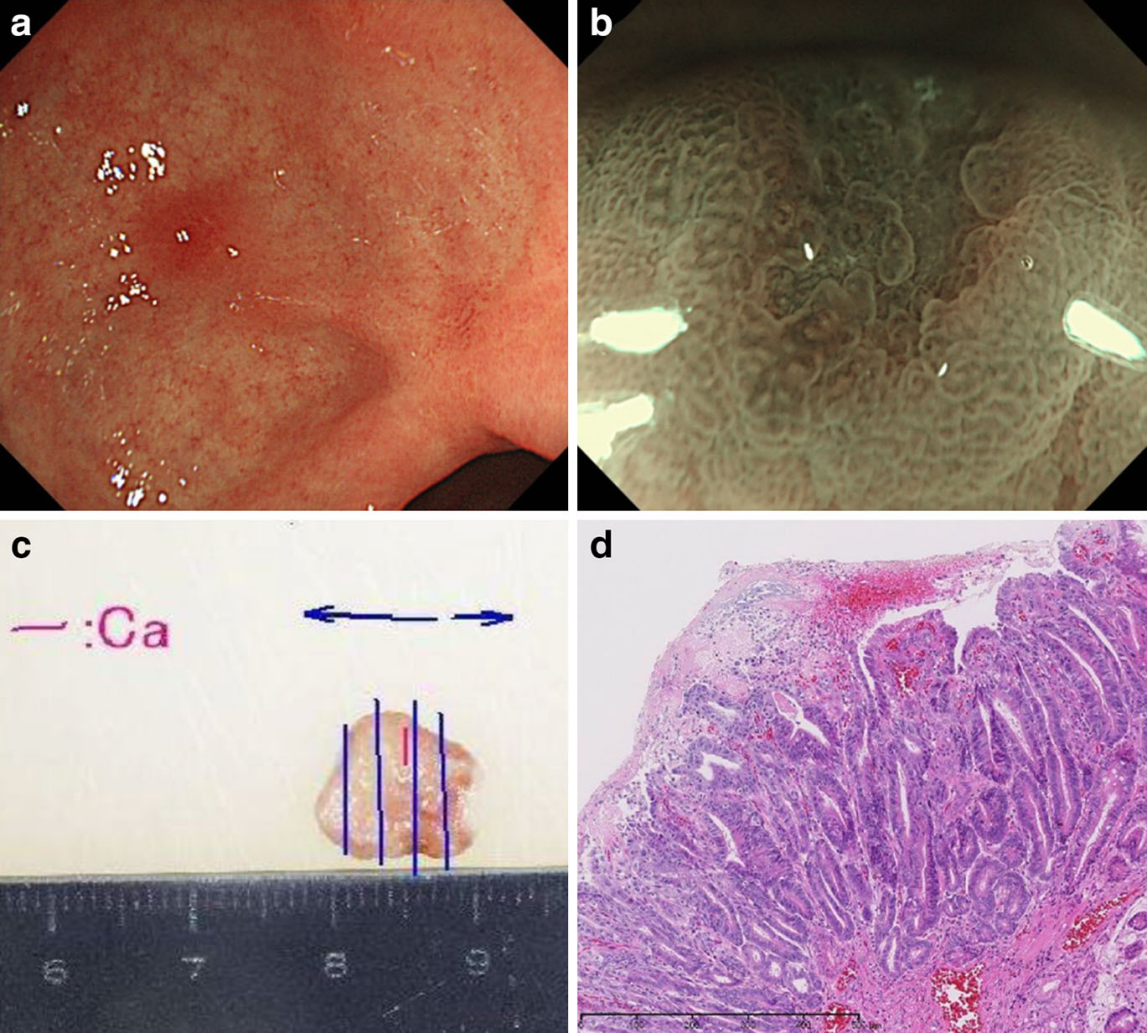
Metachronous early gastric cancer diagnosed in October 2009. **a** In October 2009, we identified a small* red* mucosal area near the scar resulting from EMR performed in 2006. **b** Using NBI, we could observe a minute depressed lesion surrounded by an irregular mucosal pattern that suggested gastric cancer. **c** We performed EMR for this lesion and extracted a 1-cm specimen on the major axis. **d** The specimen was diagnosed to be a well-differentiated adenocarcinoma of 3 × 2 mm diameter by pathological examination

**Fig. 2:**
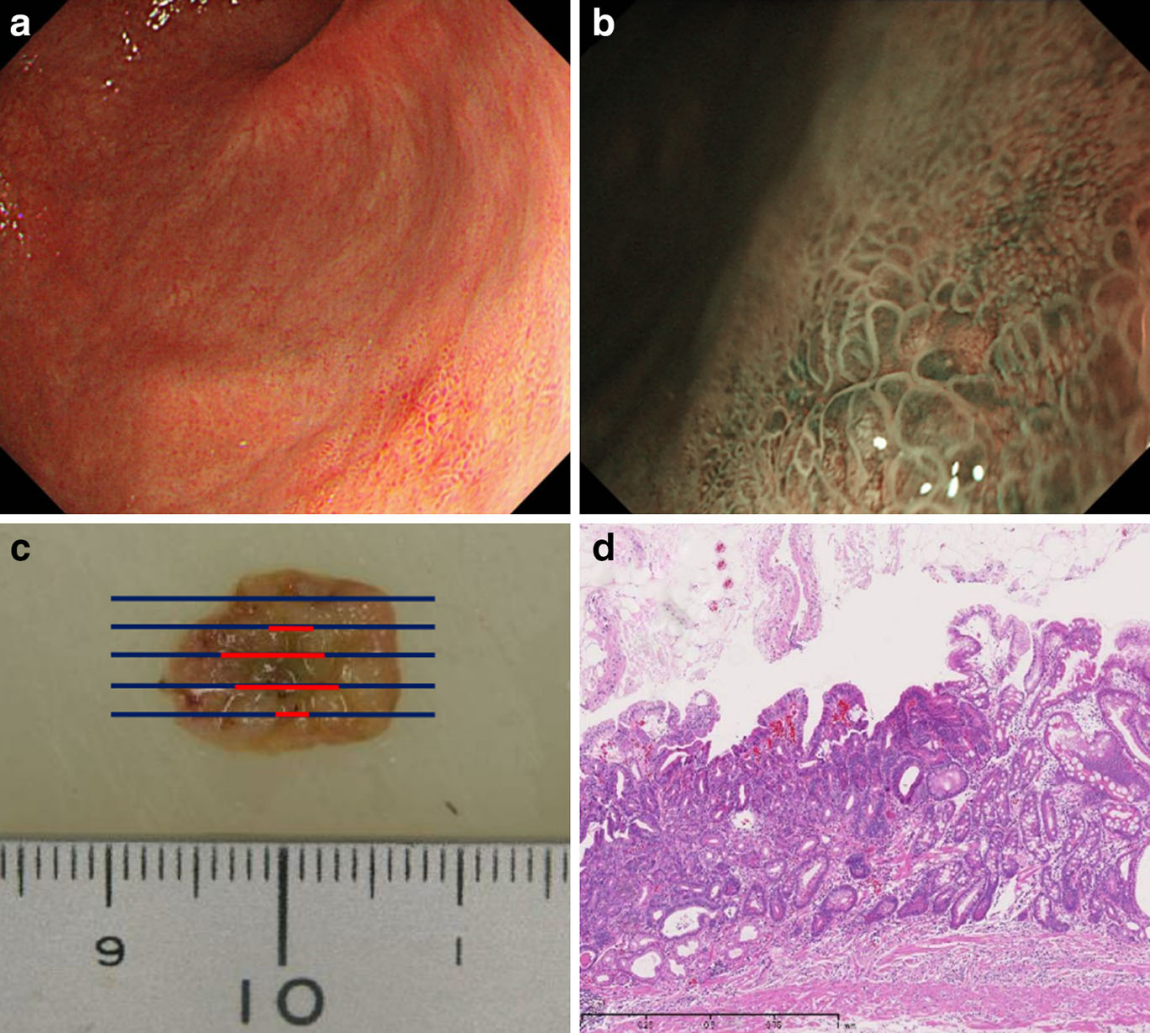
Metachronous early gastric cancer diagnosed in October 2010. **a** In October 2010, we identified a small* red* mucosa in the posterior wall of the antrum. **b** NBI examination revealed a minute depressed lesion surrounded by irregular mucosal pattern that suggested minute gastric cancer. **c** We performed EMR and extracted a 1-cm specimen on the major axis. **d** The lesion was diagnosed to be a well-differentiated adenocarcinoma of 6 × 5 mm diameter by pathological examination

Since 2007, periodic check-ups have been performed in our hospital, and in October 2009, a Stage IIc early gastric tumor (3 mm) (Fig. 1) was found at the lesser curvature of the antrum. In October 2010, a Stage IIc early gastric tumor (6 mm) (Fig. 2) was found at the posterior wall of the angulus. Both tumors were treated via EMR, and were both classified as m, tub1, HM0, VM0, ly(−), v(−), according to the histopathology findings.

**Fig. 3:**
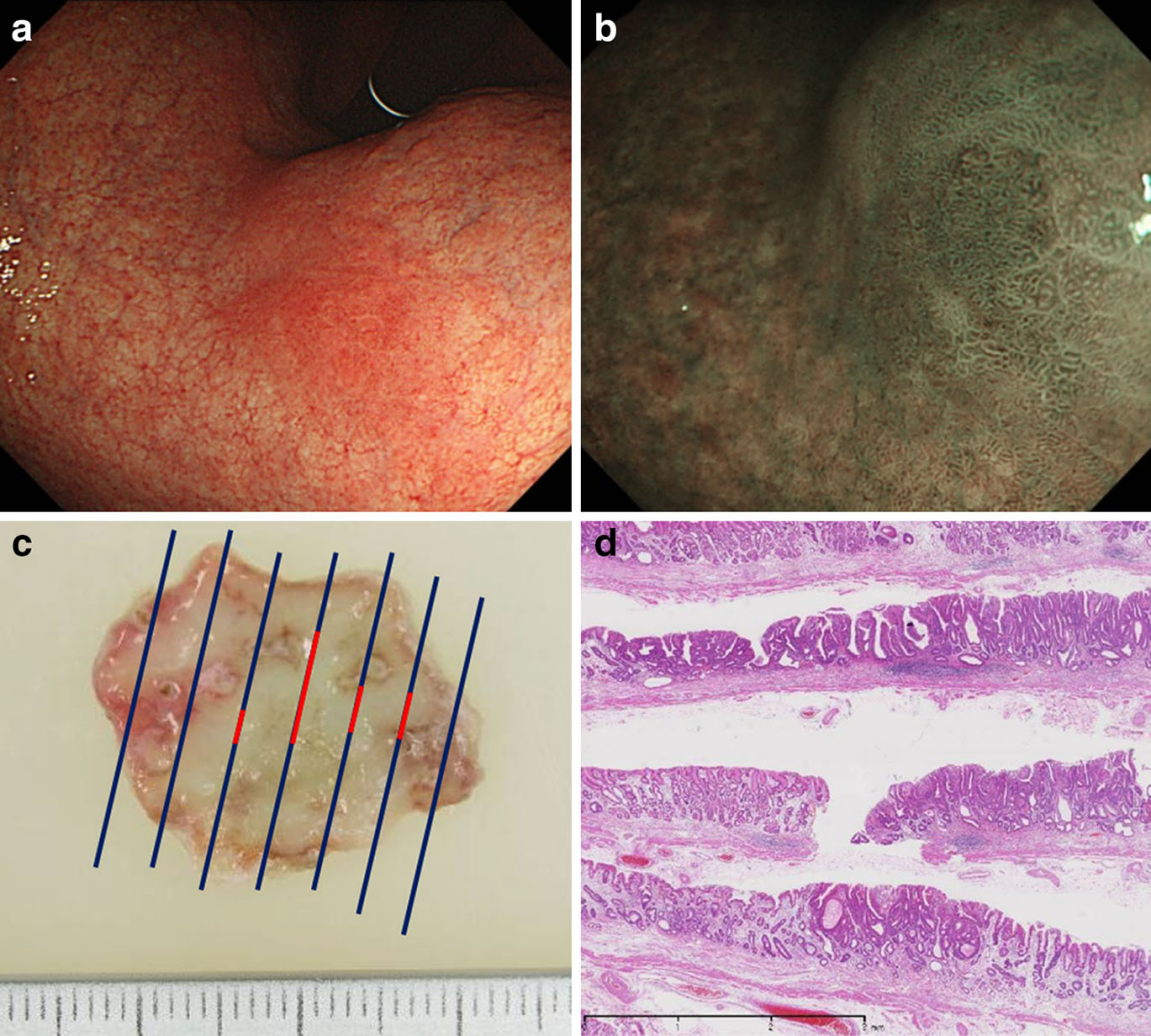
Metachronous early gastric cancer diagnosed in March 2011. **a** In March 2011, a* red* mucosa measuring 15 mm in the major axis was detected in the lesser curvature of the lower body and the visible vascular pattern of this area disappeared. **b** The demarcation line was observed around the lesion and irregular minute vessels on its surface were identified using NBI endoscopy. We diagnosed this lesion as type IIb early gastric cancer. **c** ESD was performed on this lesion and we identified a 2-cm specimen on the major axis. **d** Pathological examination revealed the lesion to be a well-differentiated adenocarcinoma measuring 10 × 7 mm

**Fig. 4:**
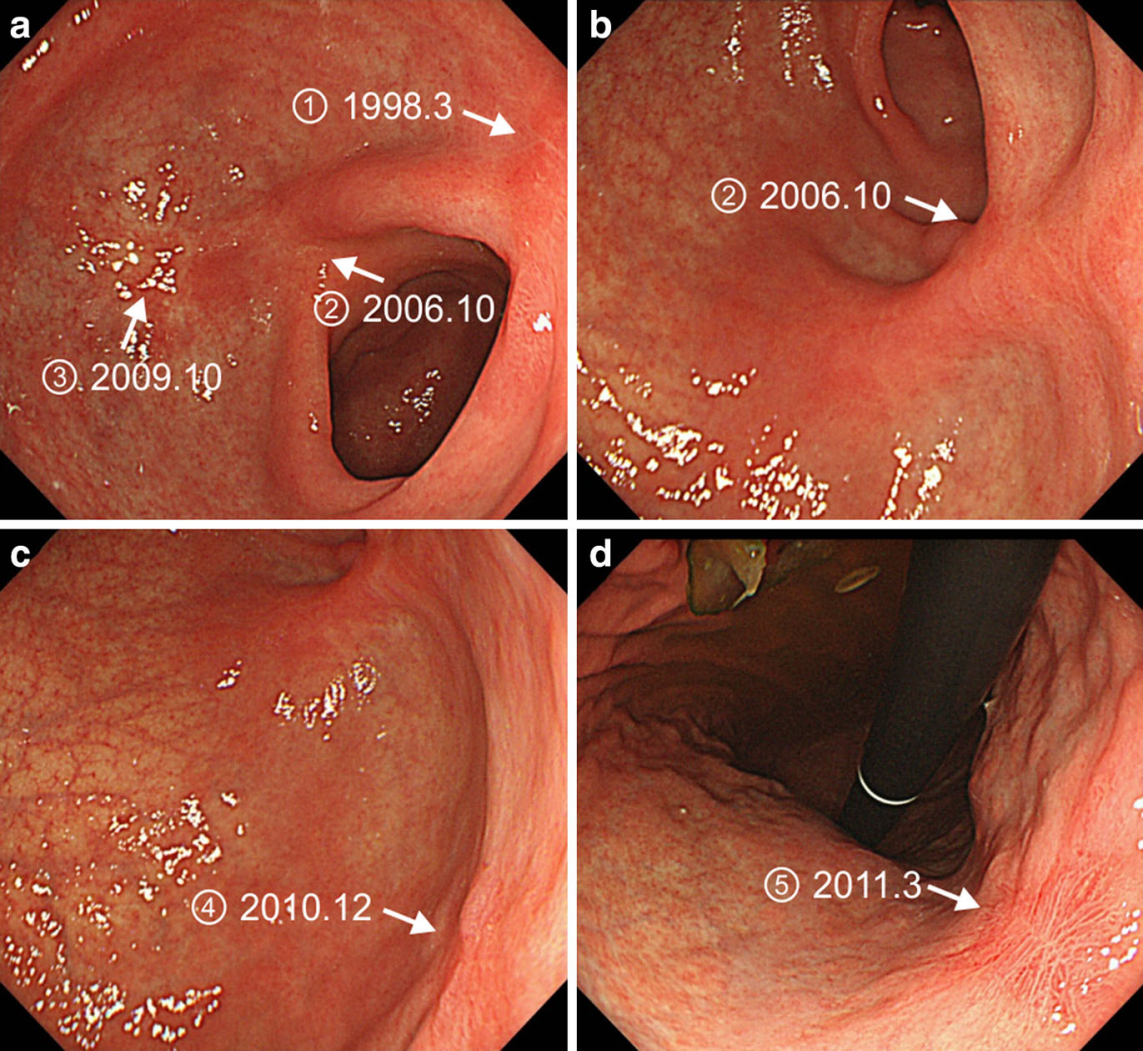
Multiple ulcer scars resulting from endoscopic resections performed between March 1998 and March 2011. **a, b** The scars after EMR performed for the second and third metachronous cancers in the antrum in 2006 and 2009, respectively. **c** The scar after EMR performed for the fourth metachronous cancer in the posterior wall of the angulus in 2010. **d** The scar after ESD performed for the fifth metachronous cancer in the lesser curvature of the middle body in 2011

In March 2011, a Stage IIb early gastric tumor (10 mm) was found at the lesser curvature of the lower gastric body and treated with endoscopic submucosal dissection (ESD) (Fig. 3). Again, the histopathology findings classified the tumor as m, tub1, HM0, VM0, ly(−), v(−), and IM(+), atrophy(+), neutrophil(−), mononuclear cell(+), *H. pylori*(−) in the background mucosa. The Kimura–Takemoto classification for endoscopic atrophy was O1, and it did not change during the endoscopic follow-up carried out yearly or half-yearly. The endoscopic treatment scars are shown in Fig. 4.

The urine antibody test performed during follow-up in 2003 and 2006 and the blood antibody and urea breath tests performed in 2011 were negative for *HP*. Since then, periodic observation with yearly upper endoscopy has been performed, and no new cancerous growths have been found.

**Fig. 5:**
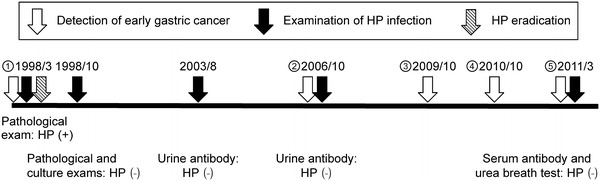
Relevant occurrences during the 13-year follow-up period. The *red arrow* shows the time of eradication of *H. pylori*. The *white arrows* show the detection times of each instance of early gastric cancer. The *black arrows* show the times and methods of examination of *H. pylori* infection during the follow-up period

**Fig. 6:**
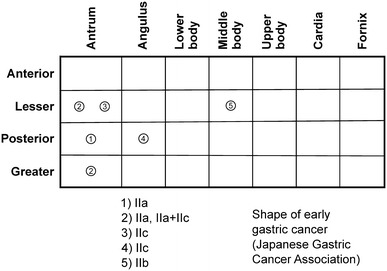
Distribution and shape of each early gastric tumor in the stomach. The location of the lesions moved to the oral side with time, and the shape of the lesions varied from an elevated type to a depressed or a flat type

Figure 5 shows the occurrences of cancer during the 13-year follow-up period, and Fig. 6 shows the locations of all the lesions.

The patient was a moderate drinker, but stopped drinking after he suffered from a head injury in a traffic accident in 2003. He is a non-smoker, does not take medicines regularly, and avoids salty foods. Except for his mother who had early gastric cancer, he had no family history of malignant disease.

## Discussion

In 1984, Marshall and Warren were able to isolate a culture of *HP* from a patient with chronic gastritis [5], and confirmed that *HP* infection is indeed the cause of various gastric illnesses, which are now understood to be infections. As a result, eradication of *HP* became the first choice of treatment for several many gastric illnesses. At first, eradication of *HP* was performed mainly for patients with peptic ulcers to prevent a relapse of the ulcers [6]. Uemura et al. indicated that gastric cancer rarely occurs in the stomach in the absence of *HP* infection, and that an infected stomach has a higher occurrence of gastric cancer during the course of observation [7]. Since then, eradication of *HP* has been extensively examined for the prevention of metachronous gastric cancer in patients who have undergone endoscopic treatment for early gastric cancer.

In 2008, Fukase et al. conducted a multi-institutional joint research to examine the effects of *HP* eradication on metachronous gastric cancer for patients who had undergone endoscopic treatment for early gastric cancer. They found a significant difference in the occurrence of metachronous gastric cancer between the eradicated and uneradicated groups, and showed that the risk of metachronous gastric cancer was reduced by up to one-third by *HP* eradication [1].

Based on these reports, eradication of *HP* has been recommended since 2009 for the diagnosis and treatment of *HP*-positive patients who have undergone endoscopic treatment of early gastric cancer; eradication of *HP* is now performed more actively from this perspective. However, even when eradication of *HP* is successful, the occurrence of metachronous gastric cancer cannot be completely prevented [2, 3].

Metachronous gastric cancer first occurred in our patient after a relatively long period, i.e., 8 years after the initial eradication, and the patient subsequently developed additional metachronous gastric tumors at 11, 12, and 13 years after eradication. These metachronous gastric cancers were found at very early stages; the tumors found at 11 and 12 years post-*HP* eradication were minute, i.e., barely detectable by magnifying endoscopy combined with narrow band imaging (NBI). Therefore, it is unlikely that these metachronous gastric tumors developed simultaneously with the first gastric cancer or immediately after the eradication of *HP*, and we speculate instead that they developed on the gastric mucosa from where the *HP* infection was removed at a later time point.

Some reports mention that the occurrence rate of metachronous gastric cancer does not decrease after *HP* eradication [8, 9], whereas the results of other studies indicate that the rate of occurrence is indeed decreased, with the decrease ranging from 1.5 % [10] to 14.6 % [11]. Thus, the types of cases that develop gastric cancer after eradication of *HP* and their differences from those that do not, are topics that need further investigation.

Take et al. [4] divided patients with peptic ulcers into 3 groups according to the levels of gastric mucosal atrophy and compared the rate of gastric cancer recurrence after eradication of *HP* in each group. Interestingly, they found that stronger levels of atrophy correlated with a more frequent occurrence of gastric cancer. For the examination of *HP* eradication after endoscopic treatment of early gastric cancer, Chon et al. [12] compared patients who developed metachronous atypical epithelium and those who did not. They found a significant difference in the degree of intestinal metaplasia on the gastric mucosa between the groups, and hence suspected that the degree of intestinal metaplasia, i.e., the level of gastric mucosal atrophy, affected the development of metachronous gastric cancer after *HP* eradication. Maehata et al. [13] also examined potential factors related to the development of metachronous gastric cancer after eradication of *HP* using multivariate analysis and indicated that an observation period of >5 years and a high level of mucosal atrophy were independent factors for metachronous gastric cancer occurrence.

Based on these studies, we believe that the development of gastric cancer after eradication of *HP* appears to be influenced largely by the presence and degree of gastric mucosal atrophy at the time of *HP* eradication, and regular observation after eradication should therefore be considered. Even though in this case the degree of intestinal metaplasia of the resected specimen by ESD was not remarkable, endoscopic atrophy already existed in the area of the fundic gland when the first lesion was found and the degree of atrophy did not change during the observation period. Thus, mucosal atrophy was identified as an important marker for metachronous gastric cancer. However, whether observation of patients who undergo *HP* eradication for peptic ulcers or chronic gastritis and patients who undergo eradication after endoscopic treatment for early gastric cancer should be performed at the same interval is an issue that must be addressed in the future. The appropriate observation period for each patient must be established while considering the burdens to the patient and from the medical economic perspective.

Watari et al. [14] indicated that patients with gastric cancer who developed metachronous gastric cancer after eradication of *HP* showed higher rates of microsatellite instability and a colonic phenotype (Das-1 protein expression), which are biomarkers for intestinal metaplasia. Hence, they pointed out that cases with intestinal metaplasia after eradication of *HP* may have a higher risk of developing metachronous gastric cancer.

In Japan, the treatment to eradicate *HP* for *HP*-related chronic gastritis, which affects a high proportion of the population, has been covered by health insurance since February 2013. Therefore, we suspect that the number of reported cases undergoing *HP* eradication has increased drastically since this time. In the future, it will be necessary to investigate whether patients undergoing *HP* eradication due to peptic ulcers or chronic gastritis and after the treatment of early gastric cancer, should be observed at the same intervals and to determine the appropriate length of long-term observation. Thereafter, it will be necessary to develop a useful clinical index to set up an appropriate observation interval for each case.
